# The Etiology of Auditory Hallucinations in Schizophrenia: From Multidimensional Levels

**DOI:** 10.3389/fnins.2021.755870

**Published:** 2021-11-11

**Authors:** Xu Shao, Yanhui Liao, Lin Gu, Wei Chen, Jinsong Tang

**Affiliations:** ^1^Department of Psychiatry, Sir Run Run Shaw Hospital, Zhejiang University School of Medicine, Hangzhou, China; ^2^RIKEN AIP, Tokyo, Japan; ^3^Research Center for Advanced Science and Technology, The University of Tokyo, Tokyo, Japan

**Keywords:** auditory hallucination, DTI, EEG, fMRI, genetics, schizophrenia

## Abstract

Enormous efforts have been made to unveil the etiology of auditory hallucinations (AHs), and multiple genetic and neural factors have already been shown to have their own roles. Previous studies have shown that AHs in schizophrenia vary from those in other disorders, suggesting that they have unique features and possibly distinguishable mechanisms worthy of further investigation. In this review, we intend to offer a comprehensive summary of current findings related to AHs in schizophrenia from aspects of genetics and transcriptome, neurophysiology (neurometabolic and electroencephalogram studies), and neuroimaging (structural and functional magnetic resonance imaging studies and transcriptome–neuroimaging association study). Main findings include gene polymorphisms, glutamate level change, electroencephalographic alterations, and abnormalities of white matter fasciculi, cortical structure, and cerebral activities, especially in multiple regions, including auditory and language networks. More solid and comparable research is needed to replicate and integrate ongoing findings from multidimensional levels.

## Introduction

Auditory hallucinations (AHs) are defined as experiences that without an external stimulus, individuals perceive voices as distinct from their own thoughts, whether the voices are familiar or not (American Psychiatric Association, 2013). With nearly 10% of lifetime prevalence rate among the general population (Maijer et al., 2018), this debilitating symptom occurs among healthy population, as well as people with various clinical conditions such as psychiatric diseases (including schizophrenia, mood disorders, dissociative disorders, etc.), neurological diseases, and hearing impairment (Laroi et al., 2012). AHs are most commonly found in major psychotic disorders, with the lifetime prevalence rate of 60-80% in schizophrenia spectrum disorders (Lim et al., 2016), and 1-year prevalence rate of 50-70% in schizophrenia specifically (Bauer et al., 2011; Waters et al., 2014). AHs are a main positive symptom of schizophrenia (American Psychiatric Association, 2013) and can bring severe damage to one’s mental health, for instance, increasing depressive symptoms (Chiang et al., 2018) and leading to suicidal ideation or attempt (Koyanagi et al., 2015).

As to the etiology, the past decades have witnessed a rapid growth in clinical studies investigating the genetic and neural substrates for AHs generally and the verbal type specifically [auditory verbal hallucinations (AVHs)] in schizophrenia. Notwithstanding, the possible mechanism remains unclear, and the existing findings are divergent to some extent. A comprehensive analysis of the ongoing studies will help depict a clearer picture of what current science knows about AHs in schizophrenia. Thus, in this review, we summarize the results from previous research, especially in the field of genetics, neurobiology, and neuroimaging. And we mainly focus on “trait” studies conducted in schizophrenia, that is, schizophrenia with auditory (verbal) hallucinations were compared to patients without the symptom instead of healthy controls. Another type of research, “state” studies that compare on-the-state period to off-the-state period within the same patient group, is also included in the respective sections. At the end of this review, we further discuss the limitations of previous studies and propose several suggestions for ways forward.

## Genetic Research

### Genetic Factor

It is well-known that gene and its interaction with environment play an important role in the development of psychiatric symptoms or disorders. And researchers have been investigating how genetic and environmental factors are linked to AHs.

Most studies were conducted among schizophrenia patients, considering the frequent occurrence of AHs in this population. Abundant studies showed that DNA variations in the *cholecystokinin A receptor* (*CCK-AR*) gene contributed to the formation of schizophrenia and AHs (Wei and Hemmings, 1999; Tachikawa et al., 2001; Sanjuan et al., 2004; Toirac et al., 2007). Schizophrenia carrying *glutathione S-transferase* (*GST*) *A1^∗^B* allele had more severe AHs than non-B carriers (Spalletta et al., 2012). Rajasekaran et al. (2016) found an association between (*human leukocyte antigen-G*) *HLA-G* 14-bp Ins/Ins genotype and lifetime presence of third person AHs, and this association was more significant in males with schizophrenia. The genotype frequency of *matrix metalloproteinase 1* (*MMP1*) single-nucleotide polymorphism (SNP) rs470558 was reported to be associated with AHs, and its A allele frequency was higher in schizophrenia with AHs (SZ-AH) than schizophrenia without AHs (SZ-non-AH) (Kim et al., 2012). *Dystrobrevin binding protein 1* (*DTNBP1*) gene was involved with neurotransmission regulation and neurodevelopment in schizophrenia, and its SNP rs4236167 was found to be associated with AHs generally and third-person and abusive form ones specifically (Cheah et al., 2015). One linkage and association analyses found that D8S1769, located 350 kb upstream of the 5′ end of the first exon of *neuregulin 1* (*NRG1*) gene, had significant linkage signal for SZ-AH, and the frequency of the G allele of SNP8NRG241930 was significantly higher in SZ-AH compared to healthy controls (Kim et al., 2006). Previous research on the association between *serotonin transporter* (*5-HTT*) gene and AHs has been controversial. *5-HTT* gene-linked polymorphic region (5-HTTLPR) is generated by a 44-bp deletion in the promoter site with two principal alleles, short (*s*) and long (*l*), respectively (Heils et al., 1996). At first, *l* allele was found to be associated with the frequency or the severity of hallucinations in schizophrenia (Malhotra et al., 1998). However, Sanjuan et al. (2006a) found that *s* allele was related to emotional response to AHs in schizophrenia, but not to the AH frequency. In another study, 5-HTTLPR polymorphism was also associated with the emotional response to AHs, specifically the distress caused by the symptom, but only with marginal statistical significance (Rivero et al., 2010).

### Genetic × Environmental Factor

Some other genes have been reported to predispose to AHs through the interaction with environmental factors. For instance, *forkhead box P2 gene* (*FOXP2*) was involved in the development of the neural systems mediating speech and language (Liegeois et al., 2003). Patients with abnormal *FOXP2* function showed significant underactivation in Broca’s area and other language-related cortical regions (Liegeois et al., 2003). And investigators found that *FOXP2* SNPs were associated with SZ-AH (Sanjuan et al., 2006b), but not directly with the symptom of AHs (Tolosa et al., 2010). Further study showed that SNP rs1456031 interacted with childhood parental emotional abuse to predict AVHs (McCarthy-Jones et al., 2014a). Putting these evidences together, *FOXP2* mutation might cause the symptom only in the presence of the environmental factor.

Based on previous findings, multiple genes, along with the influences of psychosocial factors, play their own part in the occurrence of AHs. But it is still unclear whether these genes function independently or they interact with each other, and how much percentage each gene makes up to the pathogenesis of AHs. Among them, *DTNBP1* seems to be the most potential gene candidate for AHs. It is a schizophrenia susceptibility gene related to regulation of glutamate level (Tang T. T.-T. et al., 2009), and its mRNA expression is lower in AH-related regions including superior temporal gyrus, hippocampus, and dorsolateral prefrontal cortex (Talbot et al., 2004; Weickert et al., 2004; Tang J. et al., 2009).

Although great joint efforts have been made to analyze data of large-scale genome-wide association studies in the population of schizophrenia to search for regulatory genes, such efforts are not yet made to investigate the genetic connection with AHs. Moreover, previous results are mainly derived according to bioinformatics methodologies, and it remains to be solved from molecular biological level how much influence individuals might receive from these genetic mutations.

### Transcriptional Factor

How gene is expressed in the cerebral area offers a clearer picture of genetic function on human brain. And an increasing amount of studies has investigated differentiated gene expression in specific brain areas using postmortem or database of schizophrenia. ATPase type 13A4 (ATP13A4) gene (verbal and social interaction skills) expression was up-regulated in Broca’s area of schizophrenia (Gibbons et al., 2020). Expressions of genes related to cell–cell adhesion, synaptic transmission, and neural excitability were enriched in the prefrontal cortex (Pergola et al., 2019). In dorsolateral prefrontal cortex, to be further, expressions of genes related to regulation of cell survival and growth as well as response to external stimuli (Petralia et al., 2020), related to learning and memory processes (Ohayon et al., 2020), and related to immune-related functions (Enwright and Lewis, 2021) were up-regulated, whereas expressions of genes related to mitochondrial function (Enwright and Lewis, 2021); related to neurotransmitter release modulation (Tao et al., 2020); related to cell proliferation, differentiation, and transformation (Huang et al., 2019); and related to neuronal homeostasis and intracellular signaling (Petralia et al., 2020) were down-regulated. Oxytocin receptor (social cognition) mRNA was down-regulated in the temporal cortex (Uhrig et al., 2016), and sodium-dependent MI transporter-1 (SLC5A3) gene (metabolic precursor regulation) expression was up-regulated in the superior temporal cortex (Vawter et al., 2019).

There are scattered reports about gene transcription concerning AHs only using peripheral blood samples. The mRNA level of *mitochondrial complex I gene* (NDUFV2) was found to be positively correlated with both overall and positive symptoms in the first-episode schizophrenia patients (Akarsu et al., 2014). Complement 4A (C4A) mRNA expression was found to be positively correlated with positive symptoms in schizophrenia, specifically the presence and severity of delusions (Melbourne et al., 2018). Using gene expression profiling from schizophrenia and related disorders, four genes were found to decrease in high hallucinatory state (Fn1, Rhobtb3, Aldh1l1, Mpp3), and three genes were found to increase (Arhgef9, Phlda1, S100a6) (Kurian et al., 2011). Only one study used gene expression profiling of postmortem brain samples from prefrontal cortex and found that plexin B1 (PLXNB1) expression was decreased in SZ-AH compared to SZ-non-AH (Gilabert-Juan et al., 2015). The different expression of PLXNB1 might be a signature of the hallucinatory endophenotype in schizophrenia.

Therefore, cerebral transcriptomic study of AHs in schizophrenia is scarce. As the importance is increasingly attached to transcriptomics, this research field leaves much to be exploited.

## Neurophysiological Research

### Neurometabolic Study

Magnetic resonance spectroscopy (MRS) has been an effective tool to measure cerebral metabolic level of targeted substance and has been used to investigate how certain neurochemicals might affect the occurrence of AHs. For example, as to the measurement of Glx level (composite of glutamate and glutamine), schizophrenia with AVHs (SZ-AVH) had higher Glx level than schizophrenia without AVHs (SZ-non-AVH) in the left lateral prefrontal region (Curcic-Blake et al., 2017), although schizophrenia group generally had lower Glx level than the control group in the temporal and frontal areas (Hugdahl et al., 2015; Curcic-Blake et al., 2017). Besides, AH severity was reported to positively correlate with Glx level both in the frontal and temporal regions (Hugdahl et al., 2015). Glutamate excitatory function is usually balanced by γ-aminobutyric acid (GABA) inhibitory function (Carlsson et al., 2001). Therefore, the glutamate-GABA excitatory–inhibitory imbalance could lead to the development of AHs (Hugdahl and Sommer, 2018). Further, Hjelmervik et al. (2019) found in a larger sample that Glx level was positively correlated with AVH in the left superior temporal gyrus and negatively in the anterior cingulate cortex, but they failed to find any significant result for GABA level. Consequently, they proposed that compared to Glu–GABA imbalance within regions, Glu–Glu imbalance between regions was more plausible especially in the frontal and temporal regions. Therefore, studies above have shown that glutamatergic metabolites serve as a mediating factor in AHs.

Other neurochemical studies remain scarce. N-acetyl-aspartate/choline (NAA/Cho) ratio in the right thalamus was lower in SZ-AH, relative to SZ-non-AH or healthy controls (Martinez-Granados et al., 2008). Besides, NAA level was decreased in the hippocampus in schizophrenia during the episode of AHs (Heckers, 2001) and was also reported to be negatively correlated with the duration of positive symptoms (Theberge et al., 2003). Moreover, phospholipids (phosphomonoesters and phosphodiesters) and energy metabolism (adenosine triphosphate, inorganic phosphate, and phosphocreatine) in left superior temporal gyrus were both positively correlated with AH severity in SZ-AH (Nenadic et al., 2014).

Based on existing findings, the association between glutamate level and AHs is relatively solid, but whether GABA level has a role remains inconclusive. Interregional Glu–Glu imbalance rather than intraregional Glu–GABA imbalance is more suitable at present. Findings of other neurochemical and neurometabolic studies remain scarce. It is worthwhile to finding out in future investigations how chemical substance in the brain and their metabolism influence hallucinatory activities of schizophrenia.

### Electroencephalogram Study

The forward model has been illustrated in many studies and can be applied in the auditory system (Wolpert and Miall, 1996; Heinks-Maldonado et al., 2007). When a sound initiates, the auditory feedback can be predicted by the efference copy of the motor command. This corollary discharge is compared with the actual auditory feedback. If the sound is self-generated, the predicted auditory feedback often matches the actual one; the sensory input is suppressed, leading to a dampened auditory experience. If the sound is externally produced, the predicted auditory feedback often contradicts with the actual one, leading to no suppression of the auditory response. During self-generated vocalizations, neural discharges in a majority of auditory cortical neurons are suppressed, and the suppression precedes the onset of vocalizations (Eliades and Wang, 2003), and this suppression helps distinguish between internally and externally initiated sensations. Event-related potential (ERP) results revealed that in healthy controls, N1 to self-voice feedback was dampened compared to alien voice (Heinks-Maldonado et al., 2007). Phase coherence of prespeech electroencephalogram (EEG) was found to be related to reduced speech-onset N1 potentials, showing prespeech neural synchrony suppressed subsequent responsiveness to self-spoken sound (Ford et al., 2007). However, hallucination predisposition in healthy controls might affect voice discrimination and recognition (Pinheiro et al., 2019). These evidences indicate that people with AHs could not recognize self-generated voice correctly, and they displayed improperly high response to it, as the result of which the forward model was damaged.

Schizophrenia is characterized by the disturbance of sensory gating mechanism that filters out extraneous stimuli from meaningful sensory inputs to focus attention (Freedman et al., 1987). And auditory sensory gating is one way of directly measuring auditory perceptual abnormality, and it assesses modulation of incoming auditory information 50 ms into cortical processing (Thoma et al., 2017). At 50 ms comes a positive wave, P50 potential, as the largest initial cerebral response to an auditory stimulus (Javitt and Freedman, 2015). Sensory gating deficit has been frequently found in schizophrenia (Potter et al., 2006), but limited studies have explored the relationship between sensory gating deficit and AHs. Smith et al. (2013) demonstrated that AVH severity was positively associated with the extent of P50 sensory gating deficit. Faugere et al. (2016) found that schizophrenia with P50 sensory gating impairment had more severe AVH than without that impairment. For the P50, N100, and P200 components, gating ratios were higher in schizophrenia on the state of AVH than those off the state (Thoma et al., 2017). In one magnetoencephalographic study, gating ratio of the magnetic analog of P50 (P50m) in the left hemisphere was positively associated with AHs in schizophrenia (Hirano et al., 2010). These evidences suggest that sensory gating deficit directly contributed to the formation of AHs as well.

Mismatch negativity (MMN) is another EEG-derived ERP, which functions as a neurophysiological index signaling auditory processing (Naatanen et al., 2007) and reflects detection of the change of stimulus in the environment (Salisbury, 2012). Compared to SZ-non-AH, SZ-AH possessed smaller MMNs to duration deviants (Fisher et al., 2008). In SZ-AH, MMN amplitude to gap deviants was negatively correlated with the duration, loudness, and clarity of AHs (Fisher et al., 2012). Furthermore, reduced MMN amplitude was found to be directly correlated with AHs in schizophrenia with early psychosis (Rudolph et al., 2015). Considering previous studies already reported the association between reduced MMN amplitude and general hallucinatory trait in schizophrenia (Youn et al., 2003; Fisher et al., 2011, 2014; Perrin et al., 2018), it is plausible that deficits of auditory processing and external stimulus detection, indicated by MMN alterations, could contribute to the development of AHs.

Studies have found dysfunctional γ frequency (30–100 Hz) oscillations in AHs. γ-Band oscillations play a role in selecting neurons, which communicate about sensory inputs, and higher cognitive functions including perceptual organization and language processing (Uhlhaas et al., 2008). The auditory steady-state response (ASSR), as one kind of ERPs that is elicited by temporally modulated auditory stimulation, has been used to study neural synchrony in schizophrenia (O’Donnell et al., 2013). Phase locking factor of the left hemisphere source was correlated with AHs in schizophrenia during ASSR to 40-Hz γ frequency stimulation (Spencer et al., 2009). In another study, 40-Hz ASSR was found to be diminished in schizophrenia, and phase synchronization between the primary auditory cortices was positively correlated with AHs (Mulert et al., 2011). In SZ-AVH, 40-Hz EEG activity decreased left-laterally, and global measure of phase locking decreased with stimulation (Koenig et al., 2012). Induced 40-Hz γ power in the left hemisphere was correlated with AHs in schizophrenia (Hirano et al., 2015). In another, 80-Hz ASSR-BOLD (blood oxygen–level dependent) signal was positively associated with AHs in acute episode schizophrenia (Kuga et al., 2016). Interestingly, when 40-Hz ASSR was divided as early-latency and late-latency γ response, researchers did not find group differences of early or late γ activity signatures between SZ-AH and SZ-non-AH (Griskova-Bulanova et al., 2016). Apart from ASSR-related results, correlation dimension in the γ-band in the right prefrontal cortex was more chaotic in schizophrenia with treatment-refractory AHs than counterparts without (Lee et al., 2008). Therefore, despite that participants recruited and measures reflecting γ-band oscillations differ in previous studies, results could still suggest that deficit of γ oscillations in schizophrenia was related to AHs.

Alterations of other frequency spectrums and prominent spectral interactions are also reported to be associated with AHs. Compared to SZ-non-AH, SZ-AH had increased α-band coherence between the left and right superior temporal cortices (Sritharan et al., 2005). Schizophrenia with AHs also had increased α-band phase-coupling intrahemispherically and interhemispherically and increased α-band synchrony (Angelopoulos et al., 2011). Correlation dimension in the β-band in the left parietal cortex was more coherent in schizophrenia with treatment-refractory AHs than counterparts without (Lee et al., 2008). Phase coupling between theta and γ rhythms was increased in the left frontotemporal cortices during AVH (Koutsoukos et al., 2013). β1 and β2 frequency amplitude were higher in SZ-AH than SZ-non-AH (Lee et al., 2006). Also, γ frequency was correlated with β (2 and 3) frequencies in SZ-AH, and β (1 and 2) activity was enhanced in the left inferior parietal lobule and the left medial frontal gyrus in SZ-AH relative to SZ-non-AH (Lee et al., 2006).

Combining these evidences together, SZ-AH are deficient in auditory processing, including suppressing inner speech, filtering out meaningless auditory stimuli, detecting stimulus change in the environment, and proper cerebral circuitry function (especially in the primary auditory cortex).

## Neuroimaging Research

### Structural Magnetic Resonance Imaging Study

#### Diffusion Tensor Imaging Study

Compared to SZ-non-AH, SZ-AH had lower fractional anisotropy (FA) in bilateral superior longitudinal fasciculi and arcuate fasciculi (Chawla et al., 2019). Compared to schizophrenia with audiovisual hallucinations, SZ-AH showed lower white matter connectivity in the pathways connecting the hippocampal complex with visual areas including the forceps major and the inferior fronto-occipital fasciculus (Amad et al., 2014). Different correlational studies reported that FAs in bilateral arcuate fasciculus (Rotarska-Jagiela et al., 2009), bilateral superior longitudinal fasciculi (Seok et al., 2007; Shergill et al., 2007), and left anterior cingulum (Shergill et al., 2007) were positively associated with AHs in schizophrenia. Meanwhile, mean diffusivity in left superior temporal gyrus white matter was associated with AHs specifically in male schizophrenia (Lee et al., 2009).

Previous studies focusing on verbal type of AHs have yielded inconsistent results. Relative to SZ-non-AVH, SZ-AVH had higher FA in the lateral parts of the temporoparietal section of the arcuate fasciculus (Hubl et al., 2004), in the left arcuate fasciculus (Psomiades et al., 2016), and in parts of the anterior corpus callosum (Hubl et al., 2004). Paradoxically, other studies showed that SZ-AVH had lower FA in the left frontal–temporal regions involved in language networks (including left inferior fronto-occipital fasciculus and left arcuate fasciculus segments) (Curcic-Blake et al., 2015; McCarthy-Jones et al., 2015; Oestreich et al., 2016) and in tracts involved in interhemispheric language connections (including bilateral anterior corona radiata and posterior parts of the corpus callosum) (Curcic-Blake et al., 2015). Mulert et al. (2012) reported higher but Wigand et al. (2015) reported lower FA in the interhemispheric auditory fibers in SZ-AVH. In correlational analysis, Psomiades et al. (2016) found AVH positively correlated with FA in the left arcuate fasciculus, but Curcic-Blake et al. (2015) found AVH negatively correlated with FA in the left frontal–temporal regions including arcuate fasciculus segments. In addition, some studies did not find the difference of structural integrity of internal capsule (Xi et al., 2016), anterior corona radiata (Xi et al., 2016), the language pathways (Catani et al., 2011; Leroux et al., 2017; Xie et al., 2019), and the interhemispheric auditory pathways (Leroux et al., 2017) between SZ-AVH and SZ-non-AVH, although white matter integrity in SZ-AVH or SZ-non-AVH, respectively, differed from that in healthy controls.

The debate on the involvement of the intrahemispheric and interhemispheric fasciculi in the etiology of AHs thus remains open. And the verbal type might have unique abnormalities of white matter fasciculi relative to the general hallucination. Diffusion tensor imaging (DTI) studies in the past decades are relatively inadequate, and findings of previous studies have been incongruent and contradictory. Still, it can be implied that pathological fasciculus alterations in the language pathways and interhemispheric auditory pathways lead to the emergence of AHs.

#### Morphological Thickness

Structural correlates of AHs have been frequently reported in schizophrenia. Compared to SZ-non-AVH, SZ-AVH showed reduced thickness in the right Heschl gyrus (Chen et al., 2015), in the language and primary auditory areas including the Broca’s area, the Heschl gyrus, and Wernicke’s area of the left hemisphere (van Swam et al., 2012), in the bilateral postcentral gyrus (van Swam et al., 2012), and in the left middle temporal gyrus (Cui et al., 2018). Promisingly, the abnormality of the left Heschl gyrus was replicated in schizophrenia spectrum patients with AHs (Morch-Johnsen et al., 2017), and the abnormality of the left middle temporal gyrus was confirmed by the negative correlation between its cortical thickness and AH severity (Cui et al., 2018). On the other hand, SZ-AVH showed increased thickness in the frontal cortex (left insular cortex, and bilaterally anterior/posterior cingulate, and dorsal middle frontal gyrus) and parietal lobe (van Swam et al., 2012). Among all, the left middle temporal gyrus is most related to AHs and deserves closer investigation in the future, as it is a vital part of language pathways connected to other areas (Xu et al., 2015) and related to self-monitoring dysfunction (Shergill et al., 2000).

#### Morphological Volume

Comparatively, more studies have been conducted concerning structural volume. SZ-AH showed larger volumes of temporal white matter, frontal gray matter, and temporal gray matter when compared to SZ-non-AH (Shin et al., 2005), but Kubera et al. (2014) yielded contrary results when comparing SZ-AVH to schizophrenia without or in remission with AVH. Compared to schizophrenia with audiovisual hallucinations, SZ-AH showed smaller hippocampal complex (Amad et al., 2014). Compared to SZ-non-AVH, SZ-AVH showed increased volume of the right Heschl gyrus (Hubl et al., 2010), but reduced volume of the left insula (Shapleske et al., 2002). Apart from cerebral pathological changes, Cierpka et al. (2017) found that SZ-AVH had lower gray matter volume in lobule VIIIa than SZ-non-AVH, suggesting the possible involvement of cerebellum in the pathophysiology of AVH.

There have been studies investigating the direct relationship between the hallucinatory symptom and morphological volume. In a dichotic listening task, AH severity was negatively correlated with the volume of left anterior superior temporal gyrus in SZ-AH (Levitan et al., 1999). In resting state among schizophrenia, AH severity was reported to be negatively correlated with the volume of the left superior temporal gyrus (Barta et al., 1990), corpus callosum (Knochel et al., 2012), the left Heschl gyrus, left inferior supramarginal gyrus, right middle/inferior prefrontal gyri (Gaser et al., 2004), the right superior temporal gyrus, right fusiform gyrus, and left inferior temporal gyrus (O’Daly et al., 2007), as well as left inferior frontal gyrus and right postcentral gyrus (Garcia-Marti et al., 2008). On the contrary, other studies found positive correlations between AH severity and the volume of left inferior frontal gyrus (Modinos et al., 2009), bilateral superior temporal cortex (including Heschl gyrus), left supramarginal/angular gyrus, left postcentral gyrus, and left posterior cingulate cortex (Nenadic et al., 2010). Voxel-based meta-analysis found a negative correlation between AH severity and gray matter volume in the left insula or right superior temporal gyrus (Palaniyappan et al., 2012). Another meta-analysis found the severity of AVH was correlated with volume reductions in the left and marginally the right superior temporal gyri (including Heschl gyri), implicating the bilateral structural pathology of this region (Modinos et al., 2013). But Modinos et al. (2013) failed to find group difference between SZ-AVH and SZ-non-AVH. Therefore, correlational findings very much contradict with each other.

Previous structural magnetic resonance imaging (sMRI) studies were mainly conducted in participants’ resting state. Evidences have demonstrated morphological changes in certain cerebral regions among schizophrenia, and the majority shows the shrinkage rather than the enlargement of the cerebral regions including auditory and language areas. Among all, reduced volume of temporal gyri, including Heschl gyrus, is most frequently replicated in recent research. According to studies of morphological thickness and volume, it is obvious that temporal region plays the most crucial part in the pathogenesis of AHs. However, current MRI results are heterogeneous to a certain extent and are in lack of replicability, which therefore calls for further confirmation of these results in the future.

### Functional Magnetic Resonance Imaging Study

#### Cerebral Blood Flow Study

Studies across decades have shown that cerebral blood flow (CBF) contributes to the neural underpinning of AHs. “State” study showed that schizophrenia had higher blood flow in Broca’s area in auditory hallucinating state than in their resolved state (McGuire et al., 1993). Compared to SZ-non-AVH, SZ-AVH displayed increased CBF in the right superior temporal gyrus and caudate nucleus (Zhuo et al., 2017), in the left superior temporal gyrus and right supramarginal gyrus (Wolf et al., 2012). SZ-AVH also had decreased CBF in the bilateral occipital and left parietal cortices (Zhuo et al., 2017), and in the bilateral superior and middle frontal gyri and postcentral gyri, and right supplementary motor area (Cui et al., 2017a). There were also studies focusing on CBF during task mode. When generating and monitoring inner speech, SZ-AH had reduced CBF in the left middle temporal gyrus and the rostral supplementary motor area compared to SZ-non-AH (McGuire et al., 1995). During verbal memory activation, SZ-AH had increased CBF in the left basal ganglia (Busatto et al., 1995). To sum up, CBF studies have implicated increased brain activities among auditory- and language-related regions, which accords with findings in functional MRI (fMRI) studies talked about later. Still, some results are rather sporadic, thus calling for further replications.

#### Resting-State Functional MRI

The past decades have witnessed fMRI findings emerging one after another concerning the brain activities during AHs. In order to achieve definite conclusions, meta-analysis has become a powerful systematic tool. Across populations of schizophrenia-spectrum disorder, psychotic disorder, and healthy controls, meta-analyses of trait studies revealed that AVHs were frequently related to activations in the left middle and superior temporal gyrus, left postcentral and precentral gyrus, left insula, left hippocampus/parahippocampal region, right inferior frontal gyrus, and so on (Jardri et al., 2011; Kompus et al., 2011; Kühn and Gallinat, 2012; van Lutterveld et al., 2013; Zmigrod et al., 2016). The inferior parietal lobule was also frequently reported in these analyses, but the lateralization was inconsistent. Other less frequent reported regions include anterior cingulate cortex and thalamus. Generally speaking, the cerebral activations in trait studies reflect lateralization in the left hemisphere, and related regions extensively cover the language/speech, auditory, and limbic networks. On the other hand, meta-analysis of state studies revealed that AVHs were associated with activation in bilateral inferior frontal gyrus, bilateral postcentral gyrus, and left parietal operculum (Kühn and Gallinat, 2012).

Functional connectivity is used to detect the temporal correlation of the low-frequency fluctuation in the BOLD signal between regions (Fox and Raichle, 2007), and the abnormalities of interregional resting-state functional connectivity were found among different brain areas including those reported above. Compared to patients without the symptom, schizophrenia spectrum patients with AHs had higher functional connectivity of left Heschl gyrus (belonging to the primary auditory cortex) with left frontoparietal regions and lower one with right hippocampal formation and mediodorsal thalamus, and functional connectivity of the left Heschl gyrus was correlated with AH severity in regions related to language, memory, and self-monitoring (Shinn et al., 2013). SZ-AH, compared to patients with audiovisual hallucinations, had lower functional connectivity of the bilateral hippocampal complex with the medial prefrontal cortex and the caudate nuclei and had a higher one with the thalamus (Amad et al., 2014), and dysconnectivity of hippocampal subregions was also reported in SZ-AVH (Liu et al., 2019). SZ-AH, compared to SZ-non-AH, displayed enhanced functional connectivity of the bilateral nucleus accumbens with the left superior temporal gyrus, the cingulate gyri, and the ventral tegmental area, indicating that the increased activity of the mesolimbic pathway might underlie the occurrence of AHs (Rolland et al., 2015), while in terms of AVH, SZ-AVH showed higher functional connectivity in a neural circuit involving the anterior cingulate cortex, insula, and language-related areas including superior temporal gyrus and inferior parietal lobule, compared to SZ-non-AVH (Chang et al., 2017). SZ-AVH also showed thalamic–auditory cortical hyperconnectivity and auditory cortical–hippocampal hypoconnectivity, and AVH severity was positively correlated with the connectivity from Broca’s area to the auditory cortex (Li et al., 2017). Direct correlational analyses among SZ-AVH showed that AVH severity was negatively correlated with functional connectivity in the left anterior cingulate cortex, positively correlated with the left superior temporal gyrus and right lateral prefrontal cortex (Wolf et al., 2011), and negatively correlated with neural coupling between left temporoparietal junction, bilateral anterior cingulate, and bilateral amygdala (Vercammen et al., 2010).

Within the auditory network, SZ-AH had lower interhemispheric connectivity in both primary and secondary auditory cortices when compared to SZ-non-AH (Gavrilescu et al., 2010). In another study, SZ-AH was reported to have decreased functional connectivity between two regions inside the auditory network, right Heschl gyrus, and right posterior superior temporal gyrus, compared to SZ-non-AH (Guo et al., 2020). Within the language network, SZ-AVH had higher functional connectivity of bilateral Wernicke’s area with the left inferior frontal gyrus (Hoffman et al., 2011) and reduced causal interactions from the left inferior frontal gyrus to left middle temporal gyrus (Zhang et al., 2017). In addition, the auditory cortex–posterior language network involving auditory cortex and posterior language regions was more active during AVH-on periods in schizophrenia spectrum disorders, whereas occipital–temporal and medial prefrontal networks were more active during AVH-off periods (Thoma et al., 2016). Within the default mode network (DMN), SZ-AVH had lower effective connectivity from anteromedial prefrontal cortex to left inferior temporal gyrus and from posterior cingulate cortex to left cerebellum posterior lobe, inferior temporal gyrus, and right middle frontal gyrus than SZ-non-AVH (Zhao et al., 2018). Although Guo et al. (2020) did not find dysconnectivity in the DMN, they reported that SZ-AH had reduced functional connectivity of overall parietal memory network adjacent to the DMN and also reduced functional connectivity between core regions, and the latter negatively correlated with AH severity.

Interactions between multiple networks have also been reported. With independent component analysis and dual regression, Cui et al. (2017b) demonstrated AVH-related coactivation within the auditory, default mode, executive, motor, and frontoparietal networks, which were involved in auditory processing, language production and monitoring, and sensory information filtering. Alonso-Solis et al. (2015) found alterations of resting-state functional connectivity of DMN subsystems with hubs of the salience network, suggesting cross-network abnormalities related to AVH. Further, stochastic dynamic causal modeling analysis captured the link between general ongoing hallucinatory state in schizophrenia with memory-based sensory input from the hippocampus to the salience network (Lefebvre et al., 2016). In another study, Scheinost et al. (2019) put forward a potential AVH network overlying the default mode and language processing networks.

#### Task-State Functional MRI

Studies of task-state fMRI related to AHs in schizophrenia are in lack so far. During a voice recognition task, SZ-AVH had reduced functional connectivity of right superior temporal gyrus with right superior frontal gyrus (Mou et al., 2013). Within the inner speech processing network, loudness of AVH in SZ-AVH was correlated with reduced activity in bilateral angular gyrus, bilateral anterior cingulate gyrus, left inferior frontal gyrus, left insula, and left middle temporal gyrus, during a metrical stress evaluation task activating inner speech production and perception (Vercammen et al., 2011). During verbal speech perception, SZ-AVH displayed hypercoupling in auditory–motor, language processing, and DMNs compared to SZ-non-AVH (Lavigne and Woodward, 2018), and SZ-AH comorbid with other hallucinatory types displayed a hypercoupling left-dominant temporal–frontal network involving speech-related auditory and motor regions (Lavigne et al., 2015).

Currently, neuroimaging results are still insufficient to draw any decisive conclusions. That being said, fMRI has been an effective tool to offer abundant evidences, implying that neural mechanisms underlying AHs involve abnormal activation among multiple cerebral regions related to speech/language processing, auditory perception, and so on, and disordered brain connections can be found at interregional, intranetwork, and internetwork level. Matching sMRI results and abnormal activation of superior and middle temporal gyrus are replicated constantly in fMRI studies, suggesting these regions have both structural and functional abnormalities related to AHs. As to the interregional connection, functional connectivity of Heschl gyrus, superior temporal gyrus, and hippocampus region is most noteworthy. As to intranetwork and internetwork connection, deficits of the auditory network and language network are most prominent, followed by DMN.

### Transcriptome–Neuroimaging Study

Transcriptional and neuroimaging combined studies have received huge popularity in recent years, but few has been conducted related to schizophrenia, let alone AHs. Using postmortem prefrontal cortex samples of male schizophrenia and controls and high-resolution anatomical MRI with optimized voxel-based morphometry, Sanjuán et al. (2021) found low *FOXP2* (language mediation) mRNA level was associated with reduced gray matter density, and SNP rs2396753 played a part in this association. One posttranscriptional study using PET imaging to measure the α5 subtype of the GABA receptor (α5-GABA_*A*_Rs) availability found that α5-GABA_*A*_Rs protein level was reduced in the hippocampus of antipsychotic-free schizophrenia and correlated positively with total symptom score (Marques et al., 2020), which extended previous results of lower mRNA and protein levels of α5-GABA_*A*_Rs in schizophrenia (Duncan et al., 2010; Beneyto et al., 2011). These evidences provided moderate support for central GABA hypofunction underlying the pathophysiology of clinical symptoms, albeit no direct connection with AHs. Only two studies in latest years did belong to transcriptome–neuroimaging association study of AHs. Using high-throughput RNA sequencing and resting-state fMRI data, expression of an lncRNA-mRNA network centered by lncRNA MSTRG.96171.1 was upregulated in SZ-AVH relative to SZ-non-AVH, and functional connectivity of DMN regions was positively correlated with AVH severity and MSTRG.96171.1 expression, respectively (Zhu et al., 2019). Furthermore, Yu et al. (2020) replicated the upregulated expression of this interactive network, and the positive correlation between functional connectivity of similar DMN regions and this lncRNA expression. These two pilot studies offered a clue for coexisting transcriptional and neuroimaging alterations related to AVH.

Up to present, seldom has focused on “trait” studies of SZ-AH measuring the direct relation between cerebral expression of AH-related genes and neuroimaging features. Transcriptome–neuroimaging association studies are urgently required in the future to hopefully better the understanding of endophenotypes of AHs from both molecular and imaging angles.

## Neurocognitive Model

Various models of AHs have been put forward combining evidence of all levels. Generally, there are two main models of the hallucinatory pathogenesis concerning perceptual beliefs and inner speech.

According to the computational model of perception (Fletcher and Frith, 2009; Powers et al., 2017), perceptual beliefs, originating from prior experiences, influence one’s sensation together with the actual sensory input, and hallucinations occur when the beliefs cause a percept without actual stimuli. Usually, perceptual beliefs are updated when prediction error occurs, which is the discrepancy between what is expected and what happens. Nevertheless, this mechanism has been demonstrated abnormal in schizophrenia (Corlett et al., 2007). In addition, hallucinating psychosis, regardless of the diagnosis, is less sensitive to the changes in contingency so as to have rigid perceptual beliefs and weighs more on perceptual beliefs than on actual sensory input, compared to non-hallucinating counterparts (Powers et al., 2017). Therefore, pathological perceptual beliefs may result in sensory perception even without objective stimuli, which further contributes to the formation of AHs among schizophrenia.

Another well-known model is inner speech model, which suggests that AHs arise on condition that inner speech (thinking in words) is perceived as someone else’s, and this condition results from the deficits of self-monitoring (Frith and Done, 1988). Self-monitoring refers to one’s ability to distinguish sensations evoked by one’s own actions from those by external factors (Allen et al., 2007). This process is achieved, as what is mentioned earlier about the forward model, by the comparison between the corollary discharge and the actual auditory outcome to induce different neural responses to internally or externally generated motor action. Abnormal cerebral connectivity impairs the transmission of the corollary discharge in the forward model, resulting in the deficits of self-monitoring among schizophrenia (Stephan et al., 2009; Nazimek et al., 2012). Inner speech, in this case, could also be viewed as a motor action and is accompanied by a corollary discharge (Jack et al., 2019). Thus, failure to executive the function of self-monitoring leads to the misinterpretation of inner speech as if evoked by external source.

There have been other less popular models. The aberrant memory model postulates that failure to inhibit recalling and unintendedly activation of the memory system brings past traumatic memories to consciousness and generated unexpected intrusive thoughts. Early time trauma is closely connected to later life AVH. Consequently, memories that appear out of context contribute to the sensation of “otherness” and authorship from external side and then cause AVH (Tracy and Shergill, 2013). According to the spontaneous neural activity model (also called resting-state hypothesis), the occurrence of AVH is due to enhanced resting-state activity in the auditory cortex, aberrant modulation of the auditory cortex by anterior cortical midline regions (part of the DMN), and neural confusion between auditory cortical resting-state changes and stimulus-induced activity (Northoff and Qin, 2011). According to the expectation–perception model (related to the self-monitoring mechanism), prefrontal regions are normally responsible for prediction and expectation of sensory input. As AHs happen, anatomical abnormalities in neurons, imbalance of neurotransmitter, and dysfunction of auditory cortex contribute to the deficient processing of prediction error, as a result of which the prediction from prefrontal regions becomes so unconstrained and vague that random fluctuations in spontaneous activity enhance the signal input and becomes a conscious percept in auditory cortex in the absence of external input (Nazimek et al., 2012).

## Conclusion

### Findings From Multidimensional Levels

**FIGURE 1:**
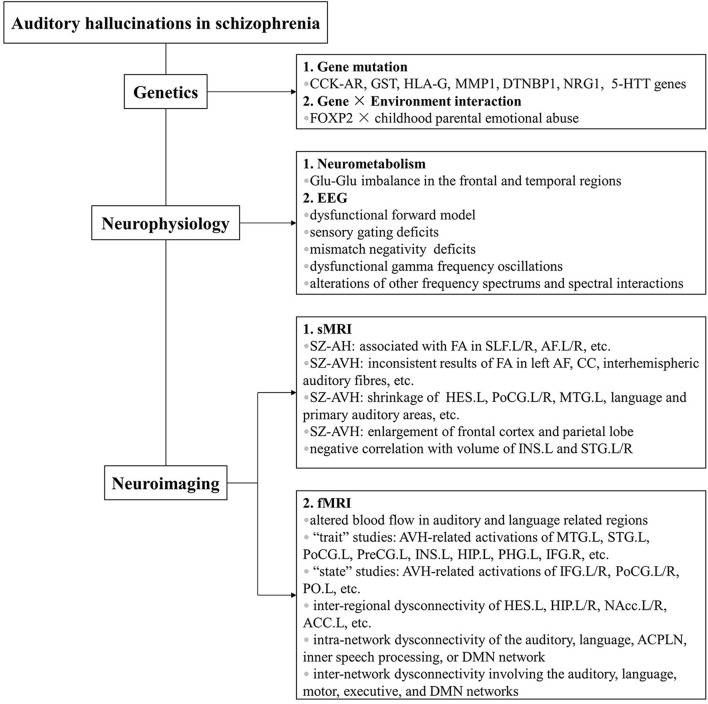
Etiology of auditory hallucinations in schizophrenia from multidimensional levels. Previous work has utilized methods of various kinds, including genetic, neurophysiological, and neuroimaging studies. Main findings are summarized in the figure. Abbreviations: CCK-AR, cholecystokinin A receptor; GST, glutathione S-transferase; HLA-G, human leukocyte antigen-G; MMP1, matrix metalloproteinase 1; DTNBP1, dystrobrevin binding protein 1; NRG1, neuregulin 1; 5-HTT, serotonin transporter; FOXP2, forkhead box P2; EEG, electroencephalogram; sMRI, structural magnetic resonance imaging; SZ-AH, schizophrenia with auditory hallucinations; FA, fractional anisotropy; L, left; R, right; L/R, bilateral; SLF, bilateral superior longitudinal fasciculi; AF, arcuate fasciculi; SZ-AVH, schizophrenia with auditory verbal hallucinations; CC, corpus callosum; HES, Heschl’s gyrus; PoCG, postcentral gyrus; MTG, middle temporal gyrus; INS, insula; STG, superior temporal gyrus; fMRI, functional magnetic resonance imaging; PreCG, precentral gyrus; HIP, hippocampus; PHG, parahippocampal gyrus; IFG, inferior frontal gyrus; PO, parietal operculum; NAcc, nucleus accumbens; ACC, anterior cingulate cortex; ACPLN, auditory cortex-posterior language network; DMN, default mode network.

With advances in our understanding of AHs, there lies an intricate fact that no single explanation so far has simply served for the full mechanism underlying these symptoms. Fruitful findings in the past decades have shown that the pathogenesis of AHs in schizophrenia has independent genetic basis and neural substrates revealed by multidimensional levels. Based on what is summarized in this review, AHs are influenced by multiple gene and gene × environment interactions from genetic level, by glutamate level imbalance from neurometabolic level, by dysfunctional forward model, sensory gating deficits, MMN deficits, dysfunctional γ frequency oscillations, and alterations of other frequency spectrums and spectral interactions from EEG level, by fasciculus alterations of white matter and morphological changes from sMRI level, and by altered cerebral blood flow, abnormal cerebral activations, and dysfunctional brain connectivity of interregion, intranetwork, and internetwork from fMRI level (summarized in Figure 1).

However, there are several aspects worthy of discussion in order to improve current research. First, small sample size, heterogeneous patients’ condition, and diversified methodologies and study designs make current evidences less easy to be replicated. One priority of future studies is to enlarge sample size and devise comparable design to allow confirmation of existing findings. Second, other types of AHs apart from the verbal type warrant more attention in the future as they are seldom studied up to present. And multimodal studies are also welcomed to combine evidences from different aspects. Third, a body of previous studies was conducted in schizophrenia with auditory (verbal) hallucinations and healthy individuals, which makes it difficult to tell whether the group differences result from the hallucinatory experience or the disease itself. As a result, “trait” study that directly compares SZ-AH and SZ-non-AH or compares SZ-AVH and SZ-non-AVH is more preferred to rule out the possible influence of the clinical state. Fourthly, “state” study that compares hallucinatory with non-hallucinatory period using self-control is tremendously scarce, probably because the ongoing hallucinatory state is relatively tricky to fully capture, and fMRI and EEG are ones of the few tools capable of distinguishing “trait” and “state” studies. Therefore, study of this kind is also wanted in the future. Last but not least, current scanty of transcriptional–neuroimaging studies of AHs calls for more endeavor in this field.

### Hypothesis 1. Schizophrenia: Auditory Hallucinations vs. Auditory Verbal Hallucinations

Although plenty of studies focus on AHs in general, there is still an abundant load of work specifically on the verbal type, AVH, probably due to the verbal type as a core positive symptom of schizophrenia. And previous research of the general and the verbal type has yielded inconsistent results.

In genetic research, gene candidates and transcriptomic studies are solely found in SZ-AH, whereas gene × environment interaction is solely found in SZ-AVH. In neurometabolic research, interregional Glu–Glu imbalance was concluded from combined evidence of both general AHs and the verbal type, and other neurometabolic studies have been mainly conducted in SZ-AH. In EEG research, sensory gating deficits are solely found in SZ-AVH, whereas other results are mainly found in SZ-AH. In sMRI research, although cerebral alterations are found from combined evidence of both SZ-AH and SZ-AVH, two groups have yielded inconsistent results. For example, regarding DTI study, “trait” studies showed SZ-AH lower FA in bilateral superior longitudinal fasciculi and arcuate fasciculi. Differently in SZ-AVH, although FA changes in other fasciculi were reported, those in superior longitudinal fasciculi were not, and results concerning arcuate fasciculi were inconclusive. For another example, regarding morphological study, scanty “trait” study in SZ-AH showed larger volumes of temporal white matter, frontal gray matter, and temporal gray matter, which did not accord with findings in SZ-AVH. In fMRI research, SZ-AVH had specific regional activation replicated by meta-analyses. While SZ-AH had enhanced functional connectivity of regions related to mesolimbic pathway, SZ-AVH of regions related to language and auditory system. While SZ-AH had dysconnectivity within the auditory network, SZ-AVH had dysconnectivity not only within the language, the inner speech processing, and the DMNs, but also between cerebral networks.

Therefore, our first hypothesis is that AVH may have independent mechanism from other types of AHs. In fact, one reason for poor replicability of current studies could be that AHs of different subtypes are treated as a whole group during research. Currently, comparative studies among subtypes of AHs in schizophrenia, although scarce, have shown that different subtypes may have distinct phenomenological features and functional brain patterns. McCarthy-Jones et al. (2014b) classified AHs into constant commanding and commenting AVH, replay AVH, own though AVH, and non-verbal AHs. One study found schizophrenia with non-verbal AHs showed higher global functional connectivity density in bilateral superior temporal gyri and lower global functional connectivity density (gFCD) in bilateral prefrontal cortex, inferior frontal lobe, and occipital lobe (Zhuo et al., 2020). These preliminary evidences have supported our hypothesis. We suggest future research should separate AHs into different groups to make subtype comparisons.

### Hypothesis 2. Auditory Hallucinations: From Genotype to Phenotype

Our second hypothesis is that AHs are heterogeneous symptoms rooted deeply in genetic background. Mediating genes cause neurophysiological alterations and structural and functional cerebral changes, which further cause distinct clinical features of AHs in schizophrenia. Based on existing genetic research, *DTNBP1* is a promising gene candidate for AHs. Its function is related to regulation of glutamate level, which is consistent with neurometabolic findings of interregional Glu–Glu imbalance (especially in the frontal and temporal regions). Its mRNA expression is lower in superior temporal gyrus and hippocampus. Regarding superior temporal gyrus, correlation between glutamate level and AHs based on neurometabolic study was found in this area. Morphological volume changes based on sMRI study and aberrant activation and functional connectivity changes based on fMRI study were also found in this area. Regarding hippocampus, structural and functional changes were also found based on sMRI and fMRI study. We hypothesized that reduced cerebral *DTNBP1* expression leads to glutamate level changes and structural and functional changes of superior temporal gyrus and hippocampus. Our hypothesis is according to current multidimensional research, but these evidences are not collected from the same patients. Research to date often utilizes a single scale, resulting in incoherent understanding of the etiology. We suggest future research use a multimode framework that uses multiscale data, from genomics, transcriptomic, neurophysiology, to neuroimaging (i.e., from genotype to phenotype). Therefore, multidimensional research conducted on one batch of patients is strongly preferred to confirm the effect of genetic variants on brain structures that contribute to AHs.

Investigations on pathogenesis of mental disorders and related phenomenology have always been a challenge hard to conquer, and AHs in schizophrenia are no exception. Although great advancement has been accomplished in the past decades, much more endeavor still needs to be made in the future. Hopefully, discovery of mechanisms underlying AHs will contribute to a deeper understanding of the essence of this symptom and will have substantial implications for clinical practice.

## Author Contributions

XS and JT conceived the study. XS completed the first draft. All authors contributed to the literature research and analyses and approved the final manuscript.

## Conflict of Interest

The authors declare that the research was conducted in the absence of any commercial or financial relationships that could be construed as a potential conflict of interest.

## Publisher’s Note

All claims expressed in this article are solely those of the authors and do not necessarily represent those of their affiliated organizations, or those of the publisher, the editors and the reviewers. Any product that may be evaluated in this article, or claim that may be made by its manufacturer, is not guaranteed or endorsed by the publisher.
